# The Impact of Novel Anti-Diabetic Medications on CV Outcomes: A New Therapeutic Horizon for Diabetic and Non-Diabetic Cardiac Patients

**DOI:** 10.3390/jcm11071904

**Published:** 2022-03-29

**Authors:** Israel Mazin, Fernando Chernomordik, Paul Fefer, Shlomi Matetzky, Roy Beigel

**Affiliations:** Department of Cardiology, The Cardiovascular Division, Sheba Medical Center, Tel-Hashomer, The Sackler Faculty of Medicine, Tel-Aviv University, Tel-Aviv 5265601, Israel; fernando.chernomordik@sheba.health.gov.il (F.C.); paul.fefer@sheba.health.gov.il (P.F.); shlomi.matetzky@sheba.health.gov.il (S.M.); beigelr@yahoo.com (R.B.)

**Keywords:** GLP1-RA, SGLT-2i, cardiovascular, heart failure, diabetes mellitus

## Abstract

It is estimated that in the past two decades the number of patients diagnosed with diabetes mellites (DM) has doubled. Despite significant progress in the treatment of cardiovascular disease (CVD), including novel anti-platelet agents, effective lipid-lowering medications, and advanced revascularization techniques, patients with DM still are least twice as likely to die of cardiovascular causes compared with their non-diabetic counterparts, and current guidelines define patients with DM at the highest risk for atherosclerotic cardiovascular disease and major adverse cardiovascular events (MACE). Over the last few years, there has been a breakthrough in anti-diabetic therapeutics, as two novel anti-diabetic classes have demonstrated cardiovascular benefit with consistently reduced MACE, and for some agents, also improvement in heart failure status as well as reduced cardiovascular and all-cause mortality. These include the sodium-glucose cotransporter-2 inhibitors and the glucagon-like peptide-1 receptor agonists. The benefits of these medications are thought to be derived not only from their anti-diabetic effect but also from additional mechanisms. The purpose of this review is to provide the everyday clinician a detailed review of the various agents within each class with regard to their specific characteristics and the effects on MACE and cardiovascular outcomes.

## 1. Introduction

It is estimated that in the past two decades the number of patients diagnosed with diabetes mellites (DM) has doubled [1]. In 2017 there were an estimated 450 million patients with DM worldwide, and this number is projected to rise to almost 700 million patients in 2045 [2]. Moreover, type 2 DM in American youth under 19 has also doubled, with a recent publication showing an increase in prevalence from 0.34 per 1000 to 0.67 per 1000 [3].

The 2017 European Society of Cardiology report states that there were about 20 million new cases of cardiovascular disease (CVD), accounting for 1133 cases per 100,000 citizens [4]. The median prevalence of DM in the same report is estimated around 7% across Europe. Multiple studies [5,6] have shown that controlling the glycemic levels in diabetic patients is associated with a marked reduction in microvascular complications. However, a number of large, well-planned prospective randomized studies comparing intensive glucose control with standard care consistently showed no benefit in reducing macrovascular complications or cardiovascular mortality. Despite significant progress in the treatment of CVD, including novel anti-platelet agents, effective lipid-lowering medications, and advanced revascularization techniques, patients with DM still are at least twice as likely to die of cardiovascular causes compared with their non-diabetic counterparts [7,8]. Indeed, a recent publication from the Swedish National Diabetes Register shows that despite a consistent reduction in the rate of cardiovascular events from 1998 to 2014, the incidence of cardiovascular death in diabetic patients still remained high, at 100 per 10,000 patient-years, and was twice for the rate for heart failure hospitalization compared with non-diabetics [9]. Accordingly, current guidelines define patients with DM at the highest risk for atherosclerotic cardiovascular disease (ASCVD) and major adverse cardiovascular events (MACE) [10,11]. Over the last few years, there has been a breakthrough in anti-diabetic therapeutics, as two novel anti-diabetic classes have demonstrated cardiovascular benefit with consistently reduced MACE, and for some agents, reduced cardiovascular and all-cause mortality [12]. Moreover, these drugs have also changed the treatment of chronic kidney disease patients, due to the close relationship between diabetic patients, cardiovascular risk, and end-stage renal disease [13]. These classes include the sodium-glucose cotransporter-2 inhibitors (SGLT-2i) and the glucagon-like peptide-1 receptor agonists (GLP-1RA). The benefits of these medications are thought to be derived not only from their anti-diabetic effect but also from other mechanisms [9].

The purpose of this review is to provide a detailed review of the various agents within each class with regard to their specific characteristics and effects on MACE and cardiovascular outcomes.

## 2. Glucagon-like Peptide-1 Receptor Agonists (GLP-1RA)

GLP-1 is an intestinal hormone that belongs to the incretin family. Their primary action is to augment insulin secretion in response to food ingestion. Additionally, GLP-1 inhibits secretion of glucagon, hence decreasing blood glucose levels through the hepatic pathway. Moreover GLP-1 reduces appetite, leading to weight loss [14]. Thus, the GLP-1 pathway has become a focus for developing pharmacological agents to treat diabetes as well as obesity. Since the biological half-life of endogenous GLP-1 is short, lasting about 2 min, modification to the active molecule is needed. Attaching Exendin-4, isolated from lizard saliva (Heloderma suspectum), and a lipid/free fatty-acid chain to the GLP-1 molecule leads to a significant reduction in GLP-1 degradation and extended half-life [15]. The GLP-1RA mimic endogenous GLP-1 activity by binding to the GLP-1R on various tissues. We focus on agents with reported CVOT outcomes, including Liraglutide, Semaglutide, Dulaglutide, and Albiglutide (Table 1 and Table 2).

### 2.1. Specific Medications

#### 2.1.1. Liraglutide

The half-life of liraglutide is 11–13 h, enabling its administration as a once daily medication. Liraglutide was the first GLP1-RA to demonstrate significant positive cardiovascular outcomes in the Liraglutide Effect and Action in Diabetes: Evaluation of Cardiovascular Outcome Results (LEADER) trial [16]. A total of 9340 patients were randomized to receive either liraglutide or placebo on top of standard care. The median follow-up was 3.8 years. The primary outcome was a combined composite of first occurrence of death from cardiovascular causes, nonfatal (including silent) myocardial infarction, or nonfatal stroke (HR 0.87; 95% CI: 0.78–0.97, *p* = 0.01 for superiority). Overall, treatment with liraglutide resulted in a significant decrease in overall death (HR 0.85; 95% CI: 0.74–0.97, *p* = 0.02) and cardiovascular death (HR 0.78; 95% CI: 0.66–0.93, *p* = 0.007), as well as the combined types of myocardial infarction (MI) (HR 0.86; 95% CI: 0.73–1.00, *p* = 0.046). However, when focusing on the different causes of MI, as well as stroke and recurrent heart failure hospitalizations, outcomes were not statistically significant. A sub-analysis of the LEADER trial showed that patients not treated with metformin had a 21% reduction in the first occurrence of the composite outcome of CV death, myocardial infarction, or stroke [17]. A post hoc analysis [18] of the LEADER trial evaluating first and recurrent MACE events showed a relative-risk reduction of 16% (HR 0.84, 95% CI: 0.76–0.93). A recently published European real-world registry including 23,000 patients propensity-score matched with DM showed that liraglutide is superior to any dipeptidyl peptidase-4 (DPP-4) inhibitor in reducing MACE [19].

#### 2.1.2. Semaglutide

Subcutaneous Semaglutide was developed as a once weekly GLP1-RA. Its long-acting property is explained by its tight attachment to albumin by a free fatty acid side chain, facilitating a pharmacological half-life of 165 to 184 h. The Trial to Evaluate Cardiovascular and Other Long-term Outcomes with Semaglutide in Subjects with Type 2 Diabetes (SUSTAIN-6) [20] included 3297 patients. The design of this trial was similar to the design of the LEADER trial, with the exception of the statistical design, which was powered for non-inferiority. Semaglutide, as compared to placebo, was proven better in lowering MACE, the occurrence of death from cardiovascular causes, nonfatal MI (including silent) or nonfatal stroke by 26% (HR: 0.74, 95% CI: 0.58–0.95, *p* < 0.001 for non-inferiority; *p* = 0.02 for superiority). Interestingly, there was a prominent decrease in non-fatal stroke by 39% (HR: 0.61; 95% CI: 0.38–0.99, *p* = 0.04) and only a trend towards a reduction in nonfatal MI (HR: 0.74; 95% CI: 0.51–1.08, *p* = 0.12). Both end-points of CV mortality (HR: 0.98; 95% CI: 0.65–1.48, *p* = 0.92) and all-cause mortality (HR: 1.05; 95% CI: 0.74–1.50, *p* = 0.79) were similar between the groups.

Oral Semaglutide was co-formulated with sodium N-(8-(2hydroxybenzoyl) amino) caprylate (SNAC) to enable gut absorption of the intact Semaglutide molecule. However, bioavailability of the oral formulation remains low, requiring daily ingestion of the medication. The Peptide Innovation for Early Diabetes Treatment (PIONEER) 6 trial [21] had a similar design to that of the SUSTAIN-6 trial and was designed to prove non-inferiority compared to placebo. Despite a higher numerical MACE in the placebo group, no statistical difference was noted (HR 0.79; 95% CI: 0.57–1.11; *p* < 0.001 for noninferiority). In an exploratory analysis, a decrease of 50% in CV death (HR 0.49; 95% CI: 0.27–0.92) and a similar reduction in all-cause mortality (HR 0.51; 95% CI: 0.31–0.84) was seen. The end-point of non-fatal stroke was not statistically significant.

#### 2.1.3. Dulaglutide

Dulaglutide consists of two modified human GLP1 molecules covalently bonded to an IgG4 heavy chain molecule. It is administered subcutaneously at a weekly dose and has a half-life of approximate 5 days. The Researching Cardiovascular Events with a Weekly Incretin in Diabetes (REWIND) trial [22] included 9901 participants who were followed up for a median 5.4 years. In this study, 31% of patients had a history of prior CV disease. There was a 12% significant reduction (HR 0.88; 95% CI: 0.79–0.99, *p* = 0.026) in the primary end point, which was defined as a composite of nonfatal MI, nonfatal stroke, and death from CV or unknown causes. The main driving force for the reduction of the primary end-point was reduction in non-fatal stroke (HR 0.76; 95% CI 0.61–0.95, *p* = 0.017). No significant benefit was shown with regards to other end-points.

#### 2.1.4. Albiglutide

Albiglutide is once weekly subcutaneously administered GLP1-RA. Its property is achieved by genetic fusion of two tandem copies of modified human GLP-1 (with 97% amino acid sequence homology to endogenous human GLP-1 fragment 7–36) to human albumin [23]. The Albiglutide and cardiovascular outcomes in patients with type 2 diabetes and cardiovascular disease (Harmony Outcomes) trial [24] enrolled a total of 9463 patients to receive albiglutide or placebo. All patients had type 2 diabetes and known cardiovascular disease. The primary end-point (in an intention-to-treat population) was the first occurrence of any component of the composite outcome, which was comprised of death from cardiovascular causes, myocardial infarction, and stroke. Patients who were treated with albiglutide had a 22% lower chance of suffering the primary outcome (95% CI: 0.68–0.90, *p* < 0.0001 for non-inferiority, *p* = 0.0006 for superiority) than those treated with placebo. Besides a 25% decrease in the outcome of fatal or non-fatal MI (95% CI: 0.61–0.90, *p* = 0.003), all other outcomes were non-significantly different to the placebo treated arm.

There are no head-to-head comparisons between the different GLP-1RA regarding clinical outcomes. Real-world data have shown that the use of GLP-1RA compared to dipeptidyl peptidase-4 (DPP-4) inhibitors is associated with lower MACE [25,26,27]. One study comparing the use of GLP-1RA to standard of care in insulin-treated patients demonstrated a reduction of 36% in MACE (adjusted HR 0.64; 95% CI: 0.42–0.98; *p* = 0.038) [28].

### 2.2. Cardio-Protection Mechanism

As shown in Table 1, GLP-1RA effects include a decrease in HBA1c, weight reduction, and improved renal outcomes [29], which are beyond the scope of the current review. The mechanism by which GLP-1RA exert their cardiovascular benefit is not clearly understood. The benefit of these medications, as demonstrated by the separation of the Kaplan–Meier curves for the primary end-point, usually appears months after initiation of therapy. This effect is similar to that seen in statin trials [30], suggesting that some of the beneficial effects may be mediated, directly or indirectly, in atherosclerosis progression and stabilization. Other potential mechanisms are attributed to blood pressure reduction as well as beneficial metabolic and renal effects, and a reduction in the need for prescription of other anti-diabetic therapies, such as insulin and/or sulfonylureas. A recent study found that both Liraglutide and Semaglutide directly decreased the atherosclerotic burden in a murine model of apo E knockout mice fed a high fat diet. This effect was mediated by a reduction in inflammation [31]. A similar effect on inflammation was found in a human model [32,33]. Other direct mechanisms on improving cardiomyocyte/cardiac fibroblast dysfunction were postulated. It was shown thar GLP-1RA improve energy balance and metabolism through multiple pathways, such as (1) preventing apoptosis through the AMPK pathway and (2) suppression of inflammation, cardiac fibrosis, and hypertrophy with GLP-1RA treatment [34].

### 2.3. Chronic Kidney Disease (CKD)

In the REWIND study [22], secondary renal outcomes, which were defined as new macroalbuminuria, a sustained decline in estimated glomerular filtration rate of 30% or more from baseline, or chronic renal replacement therapy, were evaluated. The use of dulaglutide, as compared to a placebo, was associated with a reduction in renal outcomes by 15% (95% CI: 0.77–0.93, *p* = 0.0004). In the Harmony Outcome trial [24], which evaluated albiglutide, no statistical difference was demonstrated with respect to renal outcomes, defined as a decline in the estimated glomerular filtration rate. Contrary to the above-mentioned trials, both in the LEADER trial [16] and the SUSTAIN-6 trial [20], evaluating Liraglutide and Semaglutide, respectively, there were no prespecified renal outcomes. In a recent pooled analysis of these trials, a decrease in albuminuria and eGFR decline was noted [35]. These effects were pronounced in patients with preexisting renal failure. Moreover, in an additional analysis, the use of Semaglutide, 1.0 mg weekly, was shown to provide the most benefit when compared to Liraglutide and lower doses of Semaglutide [34].

### 2.4. Adverse Outcomes/Side Effects

For GLP1-RA, the most common side effects include nausea (≈25%) and gastrointestinal discomfort (15%). These are one of the main reasons for discontinuation of these medications and are usually associated with increasing therapeutic doses, which should be done very cautiously. Slow and lenient up-titration of GLP1-RA doses helps to avoid these adverse events in some cases. Site injection reaction/sensitivity was also reported. There are reports of pancreatitis associated with GLP-1RA (0.1–2%). However, the concerning issue was a tendency toward malignancy, especially pancreatic cancer (0.1–1%). Recent meta-analysis [36] reported that no added risk was found when evaluating the current available data from the different GLP-1RA CVOTs and comparing them to DDP-4 inhibitors. Furthermore, an additional meta-analysis comparing the different CVOTs showed no difference when comparing GLP-1RA to the placebo with respect to pancreatic cancer and pancreatitis [37].

Since thyroid cell (C cells) express GLP-1 receptors, chronic activation of these cells might result in this type of cancer [38]. Thus, patients with medullary thyroid carcinoma were excluded from GLP-1RA trials and should not receive GLP-1RA. The other main adverse event is hypoglycemia, which occurs in different rates at the different trials. In the majority of the trials no difference was seen between GLP-1RA and placebo. The majority of hypoglycemic episodes were related to concomitated treatment with sulfonylureas [39].

## 3. Sodium-Glucose Cotransporter-2 Inhibitors (SGLT2i)

In the early 19th century, Phlorizin was isolated from the bark of an apple tree and was initially used to treat malaria. It was later found to have a glucosuric effect and reduce plasma glucose levels. However, a thorough understanding of the mechanism of action inhibiting glucose secretion and transport from the kidney through inhibition in SGLT-2 and SGLT-1 was established only in the 1990s [40], leading to the development of Phlorizin-like molecules. Since 2012, both the EMA and the FDA have approved several SGLT2i for the treatment of diabetes. When comparing the cardiovascular effect of SGLT2i with DDP-4 inhibitors [41] or sulfonylureas derivates [42] in real world data, SGLT2i have demonstrated superior reduction in all-cause mortality, while other outcomes were inconclusive. Further real-world observational studies have strengthened these findings [43,44,45,46]. To date, several SGLT2i have been approved: Empagliflozin, Canagliflozin, Dapagliflozin, and Sotagliflozin, which is a combined SGLT1 and SGLT2 inhibitor (Table 1 and Table 2).

### 3.1. Specific Medication

#### 3.1.1. Empagliflozin

The Empagliflozin, Cardiovascular Outcomes, and Mortality in Type 2 Diabetes (EMPA-REG OUTCOME) trial [47] was the first cardiovascular outcome trial (CVOT) to demonstrate a reduction in cardiovascular outcomes and death in diabetic patients. The primary outcome was a composite end-point of cardiovascular death, non-fatal MI, or non-fatal stroke. There was a 14% reduction in the empagliflozin group compared to placebo (HR 0.86; 95% CI: 0.74–0.99, *p* = 0.004). A significant reduction in all-cause mortality (HR = 0.68; 95% CI: 0.57–0.82, *p* < 0.001) and cardiovascular mortality was noted (HR = 0.62; 95% CI: 0.49–0.77, *p* < 0.001). No difference was seen in neurological or different MI outcomes. Surprisingly, a significant 35% decrease in heart failure hospitalizations (HR = 0.65; 95% CI: 0.50–0.85, *p* = 0.002) in the empagliflozin group was seen [48].

#### 3.1.2. Canagliflozin

Following EMPA-REG, the results of the Canagliflozin and Cardiovascular and Renal Events in Type 2 Diabetes (CANVAS) program (collaboration of the CANVAS and CANVAS-R trials) were published [49]. The primary end-point was a combination of death from cardiovascular causes, nonfatal MI, and nonfatal stroke. A 14% reduction in the primary end-point was noted (HR 0.86; 95% CI: 0.75–0.97, *p* = 0.02). However, there was no difference in all other pre-specified end-points (except for renal outcomes, which are beyond the scope of this review). On the downside, in CANVAS, patients randomized to canagliflozin had almost twice the risk of lower limb amputations (HR 1.97; 95% CI: 1.41–2.75) and increased risk for bone fractures of any kind.

#### 3.1.3. Dapagliflozin

The impressive results of the previous trials set the ground for the Dapagliflozin and Cardiovascular Outcomes in Type 2 Diabetes (DECLARE TIMI-58-) trial [50]. In this trial of diabetic patients, not only were patients with proven ASCVD included, but also patients with multiple morbidities and high cardiovascular risk (60% of total cohort). The primary safety end-point was a combination of cardiovascular death, MI, and ischemic stroke. There was no reduction in MACE (HR 0.93; 95% CI: 0.84–1.03) or all-cause mortality (HR 0.93; 95% CI: 0.82–1.04). Yet, the co-primary efficacy end-point in this trial was a combination of cardiovascular death or hospitalization for heart failure, showing a significant 17% reduction in the dapagliflozin group (95% CI: 0.73–0.95, *p* = 0.005). In a subgroup analysis of the trial, when examining only the multiple morbidity group, dapagliflozin did not show any differences with respect to the prespecified end-points. However, in the subgroup of patients with ASCVD, there was a 17% decrease (0.71–0.98) in the primary end-point of cardiovascular death or hospitalization for heart failure, but with no significant reduction in MACE (HR 0.90; 95% CI: 0.79–1.02).

### 3.2. Cardio-Protection Mechanisms

In totality, the above-mentioned trials have changed common practice, as reflected in the most recent ESC guidelines [51], which now recommend the use of SGLT2i as first-line treatment for ASCVD patients with diabetes.

At therapeutic doses, SGLT2i cause glucose excretion in the urine, increasing urinary glucose excretion and osmotic diuresis, which is associated with multiple and complex secondary effects including reduction in blood pressure and “decongestion” of the cardiovascular system (Table 1). These mechanisms might explain the early separation of the Kaplan–Meier curves for the primary end-point, which appears only weeks after the initiation of therapy. Reduction in heart failure hospitalization has now been shown in heart failure patients without diabetes, setting the stage for utilization of these medications as a heart failure medication, irrespective of diabetes status [52,53]. Proposed mechanisms for the observed effects include the novel diuretic effect [54,55], improvement in myocardial energetic mechanisms towards fatty substrate utilization [56], increased Ca^2+^ efficacy at the cell level [57], and induction of autophagy due to constant glucosuria [58,59]. Furthermore, it is postulated that the inhibition of sodium-hydrogen exchangers (NHE1 at heart and vessels and NHE3 at kidney) by SGLT2i may improve treatment of heart failure patients through beneficial effects on insulin sensitivity, improved diabetic treatment, and decrease of the sympathetic tone [60].

### 3.3. Heart Failure

The usefulness of SGLT2i for the treatment of heart failure patients has been assessed in patients with reduced ejection fraction (HFrEF) and in patients with preserved ejection fraction (HFpEF).

#### 3.3.1. Heart Failure with Reduced Ejection Fraction (HFrEF)

##### Dapagliflozin

The first trial evaluating treatment with SGLT2i for heart failure patients was the Dapagliflozin in Patients with Heart Failure and Reduced Ejection Fraction (DAPA-HF) trial, published in 2019 [52]. This was the first SGLT2i trial to include both diabetic and non-diabetic patients. Included patients had an LVEF ≤ 40%, NYHA class ≥ II, and elevated NT-proBNP levels, and had been hospitalized due to heart failure in the year prior to enrolment. The primary composite outcome was the occurrence of the first event of either worsening heart failure (hospitalization or an unplanned clinic visit) or cardiovascular death. The trial recruited 4744 patients, and the incidence of the primary composite outcome was 26% lower in the dapagliflozin group compared to the placebo group (95% CI: 0.65–0.85, *p* < 0.001). Moreover, there was a prominent decrease in the rate of heart failure hospitalization in the dapagliflozin group (HR 0.7; 95% CI: 0.59–0.83). This effect was consistent whether patients were diabetic or not. No major side effects were noted between the groups, including no events of hypoglycemia in the non-diabetic patients.

##### Empagliflozin

The Empagliflozin Outcome Trial in Patients with Chronic Heart Failure and a Reduced Ejection Fraction (EMPEROR-reduced) trial was published in late 2020 [53]. As with DAPA-HF, patients included had a LVEF ≤ 40% and NYHA class ≥ II. The primary composite end-point was cardiovascular death or heart failure re-hospitalization. Of the total 3730 patients recruited, the incidence of the primary composite outcome was 25% lower in the empagliflozin group compared to placebo (95% CI: 0.65–0.86, *p* < 0.001). Similar to the DAPA-HF trial, a 30% reduction in heart failure re-hospitalization was noted in the empagliflozin group compared to placebo (95% CI: 0.58–0.85, *p* < 0.001). However, no difference was seen between the treatment groups, either all-cause or cardiovascular death. Outcomes in this trial were, again, irrespective of diabetic status.

Regarding the composite end-point of cardiovascular death and first heart failure hospitalization, both medications showed a similar reduction of about 25%. A similar trend was observed when comparing the outcome of all heart failure re-hospitalization (first and recurrent) to the above-mentioned composite outcome [61]. In the EMPEROR-reduced trial, a 58% decrease in all-cause mortality, heart failure hospitalization, or urgent visit due to worsening heart failure was noted already 12 days after randomization [62]. In the DAPA-HF trial, a reduction of 49% in the composite outcome of cardiovascular death or worsening heart failure was noted 28 days after drug initiation [63]. Given the rapid clinical effect, initiating SGLT2i therapy during heart failure hospitalization has been advocated [64].

##### Sotagliflozin

Sotagliflozin is an SGLT2i, but with an additional active site inhibiting the SGLT1 receptor in the gastrointestinal tract. SGLT1 inhibition is postulated to decrease/delay glucose reabsorption from the gastrointestinal tract, decreasing mainly post-prandial glucose levels [65,66]. Moreover, SGLT1 is responsible for about 10% of the glucose reabsorption that is filtered through the renal proximal tubule segment 3 [67].

The aim of the recent published Sotagliflozin in Patients with Diabetes and Recent Worsening Heart Failure (SOLOIST-WHF) trial [68] was to evaluate the use of Sotagliflozin in diabetic patients with acute heart failure. Patients were enrolled during hospitalization due to acute heart failure with elevated natriuretic peptide and were hemodynamically stable with no need for inotropic support. There was no LVEF limit, and the primary end-point was cardiovascular death and heart failure hospital re-admission (first and subsequent). A 33% decrease in the primary end-point was noted in the Sotagliflozin group (95% CI: 0.52–0.85, *p* < 0.001). This trend was maintained when considering only first heart failure hospitalization (HR 0.71; 95% CI: 0.56–0.89, *p* < 0.001). When evaluating the outcome of heart failure hospitalization or urgent visit due heart failure, patients treated with Sotagliflozin had a 36% lower risk compared to the placebo (95% CI: 0.49–0.83, *p* < 0.001). No difference was noted in all-cause mortality. Interestingly, since the trial also enrolled patients with preserved LVEF, a sub-group analysis found that patients with an LVEF ≥ 50% had a 52% reduction in the HR for the primary end-point events (95% CI 0.27–0.86).

#### 3.3.2. Heart Failure with Preserved Ejection Fraction (HFpEF)

The recent results of the SOLOIST-WHF trial [68] and a post hoc analysis from the DECLARE-TIMI 58 [69] set the framework for dedicated clinical trials in patients with heart failure and preserved ejection fraction (HFpEF).

The first trial evaluating specifically patients with HFpEF was the EMPEROR-Preserved Trial [70] enrolling patients with an LVEF > 40% and NYHA ≥ II. As seen in most HFrEF trials, half of the patients were diabetic. The primary end-point was death from cardiovascular causes or hospitalization for heart failure. A 21% decrease in the occurrence of the primary end-point was observed in patients treated with empagliflozin compared to placebo (95% CI: 0.69–0.90 *p* < 0.001). The main driving event was a reduction in re-hospitalization (95% CI: 0.71, 0.60–0.83). No difference was noted in cardiovascular death. Total heart failure rehospitalizations were reduced by 27% in the empagliflozin group (95% CI: 0.61–0.88, *p* < 0.001). These results were consistent in the majority of sub-groups, including diabetic and non-diabatic patients. There was a more pronounced effect in patients with an LVEF > 60% compared to those with an LVEF of 41–59%.

The SGLT2 inhibitor dapagliflozin in heart failure with preserved ejection fraction: a multicenter randomized trial (PRESERVED-HF) [71] examined the effect of dapagliflozin in HFpEF patients with respect to symptoms, physical limitations, and exercise function as measured by the KCCQ-CS after 12 weeks. The primary outcome (improvement in KCCQ-CS) was improved in the dapagliflozin group (effect size, 5.8 points (95% CI 2.3–9.2, *p* = 0.001)). Moreover, there was an increase in the six-minute walk test for patients treated with dapagliflozin, with an effect size of 20.1 m (95% CI 5.6–34.7, *p* = 0.007). Similar results were reported for the EMPEROR-Preserved Trial [72] across the different KCCQ tertiles. The odds ratio for preventing ≥ 5-point deterioration was 0.85 (95% CI: 0.75–0.97) for the empagliflozin treated patients, resulting in a number needed to treat of 35 in order to prevent deterioration. The currently ongoing DELIVER study will evaluate the effect of dapagliflozin in patients with HFpEF [73].

#### 3.3.3. Acute Heart Failure

The effect of empagliflozin on clinical outcomes in patients with acute decompensated heart failure trial (EMPA-RESPONSE-AHF) was a randomized, double-blind, placebo-controlled, multicenter pilot study on the effects of empagliflozin on clinical outcomes in patients with acute decompensated heart failure); this was a pivotal trial for exploring the usefulness of empagliflozin in acute heart failure patients [74]. The trial included 80 acute heart failure patients randomized to either empagliflozin or a placebo. The results of this trial demonstrated good tolerance and safety of empagliflozin in the setting of acute heart failure. Although there was no improvement in dyspnea, decrease in NT-proBNP, or change in the length of hospital stay, a reduction of the combined end-point of worsening HF, rehospitalization for HF, and death at 60 days was noted (10% vs. 33.3%, *p* = 0.014).

The SGLT2 inhibitor empagliflozin in patients hospitalized for acute heart failure (EMPULSE) trial [75] further evaluated the efficacy and safety of empagliflozin. In this trial, 530 patients with acute heart failure were randomized to empagliflozin or placebo groups. The median time to initiation of empagliflozin was 3 (2–4) days from presentation. The combined end-point included a composite of death, heart failure hospitalizations, time to first heart failure hospitalization, and change from baseline in the Kansas City Cardiomyopathy Questionnaire Total Symptom Score after 90 days of treatment. Patients treated with empagliflozin had a 36% improvement in the primary outcome as compared to placebo. Additionally, when evaluating individual secondary end-points, patients treated with empagliflozin had lower mortality (4.2% vs. 8.3) and lower heart failure events (10.6% vs. 14.7). No safety issues were reported with the early initiation of empagliflozin.

### 3.4. Chronic Kidney Disease (CKD)

In the abovementioned SGLT-2i trials, renal outcomes were considered as a secondary outcome. In these trials, patients with an eGFR below 30 mL/min/1.73 m^2^ were excluded. Along with the differences in the baseline characteristics of patients between the different trials, there were also different definitions regarding renal outcomes. In the EMPA-REG trial, a post hoc analysis [76] defined renal outcomes as doubling of serum creatinine, ESRD, or renal death. The use of empagliflozin was associated with a 46% reduction in renal outcomes (95% CI: 0.40–0.75, *p* < 0.001). In the DECLARE TIMI-58 trial [50], renal outcomes that were defined as a ≥40% reduction in eGFR to a threshold <60 mL/min/1.73 m^2^, renal/cardiovascular death, end stage renal disease (which was defined as dialysis ≥ 90 days or sustained eGFR < 15 mL/min/1.73 m^2^), or kidney transplantation were reported with an HR  of 0.53 (95% CI 0.43–0.66, *p* < 0.001). In the SCORED trial [66], renal outcomes were defined as first occurrence of a sustained decrease of ≥50% in eGFR from baseline for ≥30 days, long-term dialysis, renal transplantation, or sustained eGFR of <15 mL/min/1.73 m^2^ for ≥30 days. No statistical difference was seen between the SGLT-2i and placebo groups.

In both SGLT2i heart failure trials DAPA-HF [52] and EMPEROR-Reduced [53], renal outcomes were prespecified. In the DAPA-HF, renal outcome was defined as composite outcome of a reduction of 50% or more in the estimated GFR sustained for at least 28 days, end-stage renal disease, or death from renal causes. End-stage renal disease was defined as an estimated GFR of less than 15 mL/min/1.73 m^2^ that was sustained for at least 28 days, long-term dialysis treatment (sustained for ≥28 days), or kidney transplantation. No difference was noticed between dapagliflozin and placebo (HR 0.71; 95% CI: 0.44–1.16). In the EMPEROR-Reduced trial, the composite renal outcome included chronic dialysis; renal transplantation; a sustained reduction of 40% or more in the estimated GFR; a sustained estimated GFR of less than 15 mL/min/1.73 m^2^ in patients with a baseline estimated GFR of 30 mL/min/1.73 m^2^ or more; or a sustained estimated GFR of less than 10 mL/min/1.73 m^2^ in those with a baseline estimated GFR of less than 30 mL/min/1.73 m^2^. A 50% reduction in renal outcomes was noted between patients treated with empagliflozin compared to those treated with a placebo (95% CI: 0.32–0.77).

Perhaps the most important and dedicated trial to date evaluating renal outcomes associated with treatment with SGLT2i is the Dapagliflozin and Prevention of Adverse Outcomes in Chronic Kidney Disease (DAPA-CKD) trial [77]. In this trial, which evaluated 4304 patients with or without diabetes and with an estimated GFR of 25–75 mL/min/1.73 m^2^ and a urinary albumin-to-creatinine ratio of 2000–5000, patients were randomized to therapy with either dapagliflozin 10 mg daily or placebo and were followed up for a median of 2.4 years. The primary outcome was a composite of a sustained decline in the estimated GFR of at least 50%, end-stage kidney disease, or death from renal or cardiovascular causes. It was shown that regardless of the presence or absence of diabetes, the risk of a composite of a sustained decline in the estimated GFR of at least 50%, end-stage kidney disease, or death from renal or cardiovascular causes was significantly lower with dapagliflozin than with the placebo (9.2% vs. 14.5%, HR 0.61; 95% CI 0.45–0.72, *p* < 0.001); the number needed to treat to prevent one primary outcome event was 19 (95% CI 15–27).

### 3.5. Adverse Outcome/Side Effects

The main adverse events reported with SGLT2i are the tendency to develop urosepsis or pyelonephritis (0.4%), genital mycotic infections (0.9%), and hypoglycemia (up to 1.4%), which is augmented by intake of insulin intake or sulfonylureas [78]. Additionally, as a consequence of their diuretic effects, patients, especially the elderly, can be more prone to dehydration and orthostatic hypotension. Physicians administrating SGLT2i should bear in mind to adequately adjust doses of additional diuretics administrated concomitantly when initiating SGLT2i. The early and most worrisome adverse event that was reported, and was solely associated with the use of Canagliflozin, was an increased risk of lower limb amputation. An additional major adverse event is the development of euglycemic ketoacidosis, which was reported in the different SGLT2i CVOTs to be between 0.1–0.3%; however, this is probably considered to be more common in daily practice [79]. The major predisposing risk factors for SGLT2i-associated ketoacidosis are (1) an acute stressogenic event such as reduced caloric intake due to illness or surgery and/or an acute febrile illness, (2) insulin dose reduction, (3) pancreatic disorders related to insulin deficiency (DM type I, pancreatitis, and pancreatic surgery), and (4) alcohol abuse.

## 4. Current Guidelines

Based on the current findings from the CVOTs mentioned, the European Society of Cardiology released the diabetes treatment guidelines in 2019 [51]. In these guidelines, there was a shift in the recommendations on how to treat diabetic patients with ASCVD. For drug-naïve diabetic patients, it is recommended to initiate therapy with either an SGLT2i or a GLP-1RA as the first line of treatment. For patients already treated with metformin, the guidelines recommended adding either an SGLT2i or a GLP-1RA (Class I recommendation). The recent American Diabetes Association (ADA) “Standards of Medical Care in Diabetes” publication [80] concurs with the ESC guidelines regarding the use of SGLT2i or GLP-1RA in patients with diabetes and established ASCVD (Class A recommendation). An analogous approach was recently adopted by the American College of Cardiology (ACC) [81].

Additionally, the emerging data regarding the use of SGLT2i from heart failure studies have paved the way for these medications (initially dapagliflozin) as a class I recommendation for heart failure patients, irrespective of their diabetes status, in the recent ESC heart failure guidelines [82] and in an expert consensus update published by the ACC in 2021 [83].

## 5. Non-Pharmacological Interventions

Although novel anti-diabetic medications have enhanced our ability to treat diabetic cardiovascular patients, it is of outmost importance not to neglect non-pharmacological interventions such as adherence to healthy lifestyle, including adequate physical activity, avoidance of sedentary behavior, maintaining adequate dietary habits, and adhering to weight reduction programs when appropriate. This is recommended to prevent the chronic complications associated with diabetes [84,85]. It has been well proven that for the diabetic patient, physical activity is associated with better glycemic control [86,87] and an improved quality of life [88]. Moreover, several studies have shown the association between enhanced physical activity and a reduction in mortality [89,90], the prevention of recurrent cardiovascular events [91], and a decrease in the incidence of heart failure [92] among diabetic patients.

## 6. Conclusions and Future Perspective

In less than a decade, GLP-1 RA and SGLT2i have led to a revolution in the field of medical cardiovascular care. The results of the CVOTs have given us the opportunity to adjust and tailor treatment for diabetic patients with ASCVD, as well as for those with heart failure, irrespective of diabetic status, for SGLT2i, with either reduced or preserved ejection fraction. We now eagerly await the results of ongoing clinical trials evaluating the use of GLP1-RA for non-diabetic patients with overweight or obesity [93], as well as for diabetic patients with cardiovascular disease (SOUL trial, www.clinicaltrials.gov, NCT03914326), and for trials evaluating the use of SGLT2i for diabetic and non-diabetic patients with acute coronary syndromes (EMPACT-MI, NCT04509674 and DAPA-MI, NCT04564742; www.clinicaltrials.gov) trials as well ACS and acute heart failure as DAPA-ACT HF TIMI-68 (www.clinicaltrials.gov, NCT04363697)). With their widespread use in various cardiovascular scenarios, SGLT2i have been coined “the statins of the 21st century” [94]. Both SGLT2i and GLP1-RA should be considered as first line therapy for every diabetic patient with ASCVD.

**Figure 1:**
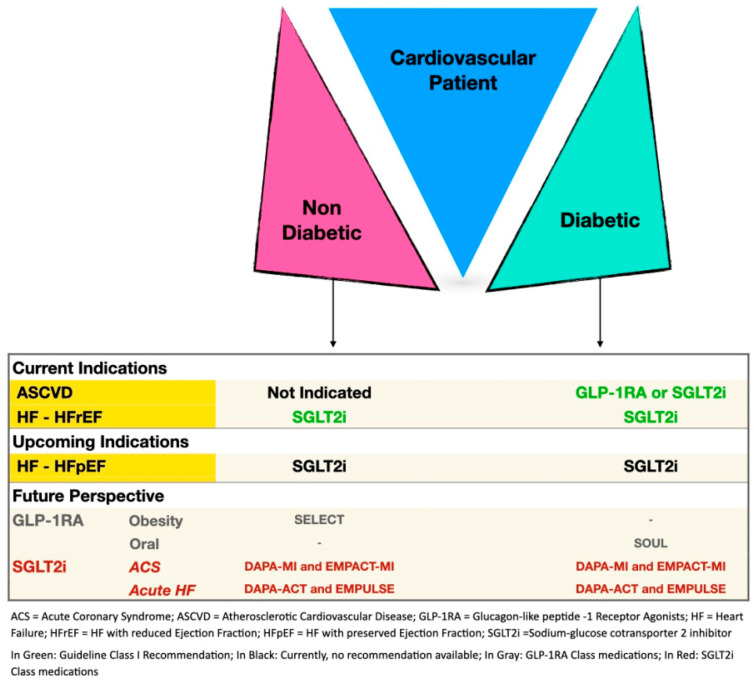
The various indications as well upcoming and future perspectives for novel diabetes medications for the cardiovascular patients, for both diabetic as well as non-diabetic, patients. ACS = Acute Coronary Syndrome; ASCVD = Atherosclerotic Cardiovascular Disease; GLP-1RA = Glucagon-like peptide-1 Receptor Agonists; HF = Heart Failure; HFrEF = HF with reduced Ejection Fraction; HfpEF = HF with preserved Ejection Fraction; SGLT2i = Sodium-glucose cotransporter 2 inhibitor.

Figure 1 title: The various indications as well upcoming and future perspectives for novel diabetes medications for the cardiovascular patients, for both diabetic as well as non-diabetic, patients.

Figure 1 legend: ACS = Acute Coronary Syndrome; ASCVD = Atherosclerotic Cardiovascular Disease; GLP-1RA = Glucagon-like peptide-1 Receptor Agonists; HF = Heart Failure; HFrEF = HF with reduced Ejection Fraction; HfpEF = HF with preserved Ejection Fraction; SGLT2i = Sodium-glucose cotransporter 2 inhibitor.

In Green: Guideline Class I Recommendation; In Black: Currently, no recommendation available; In Gray: GLP-1RA Class medications; In Red: SGLT2i Class medications.

Table 1 title: Major pharmacological effects and side-effects from cardiovascular outcome trials evaluating novel anti-diabetic medications.

Table 1 Legend: ASCVD definition = Coronary heart disease, cerebrovascular disease, peripheral vascular disease, chronic kidney disease of stage 3 or greater.

Table 2 title: Major clinical outcomes from cardiovascular outcome trials evaluating novel anti-diabetic medications.

Table 3 title: Major clinical outcomes from heart failure studies evaluating SGLT2i.

Table 3 Legend: HFrEF: Heart failure with reduced ejection fraction; HFpEF: Heart failure with preserved ejection fraction. ^&^ The primary outcome was a composite of adjudicated cardiovascular death or hospitalization for heart failure. * The primary end-point was the composite of worsening heart failure (hospitalization or an urgent visit resulting in intravenous therapy for heart failure) or death from cardiovascular causes. ^$^ Trial included both HFrEF and HFpEF patients. ^#^ Primary end-point included death from cardiovascular causes and hospitalizations and urgent visits for heart failure—total number of events.

Tables: The impact of novel anti-diabetic medications on CV outcomes: A new therapeutic horizon for diabetic and non-diabetic cardiac patients.

## Figures and Tables

**Table 1:** Major pharmacological effects and side-effects from cardiovascular outcome trials evaluating novel anti-diabetic medications.

Drug Class	Name of Anti-Diabetic Drug Evaluated, Study Name, Number of Patients Enrolled (N)	Patient Population	Effect on HBA1c	Effect on Weight	Effect on Blood Pressure	Renal Deterioration (Decrease in eGFR, Proteinuria and Dialysis)	Major Side Effects
GLP1-RA	Liraglutide vs. Placebo (LEADER) (16) (29) N = 9340 All diabetic Follow-up duration (Median): 3.8 years	(1) ≥50 years of age with ASCVD or HF NYHA II/III (2) ≥60 years or more with at least one cardiovascular risk factor: Microalbuminuria or proteinuria, Hypertension and left ventricular hypertrophy, Left ventricular systolic or diastolic dysfunction, or Ankle–brachial index < 0.9	↓ −0.40%	↓↓ −2.3 kg	↑ Systolic 1.2 mmHg Diastolic 0.6 mmHg	Prevent deterioration	Gastrointestinal disorders, Acute gallstone disease, ↑ Heart Rate
	Semaglutide vs. Placebo (SUSTAIN-6) (20) (29) N = 3297 All diabetic Follow-up duration (Median): 2.1 years	(1) Age ≥ 50 years with ASCVD (2) Age ≥ 60 years of age with cardiovascular risk factors only (as above)	↓↓ (−1.1%) to (−1.4%)	↓↓↓ (−3.6 kg) to (−4.9 kg)	↓ Systolic −3.4 mmHg to −5.4 mmHg /Diastolic −2.2 mm/Hg to −2.8 mmHg	Prevent deterioration	Gastrointestinal disorders, ↑ Heart Rate, Retinopathy
	Semaglutide (Oral) vs. Placebo (PIONEER-6) (21)(29) N = 3183 All diabetic Follow-up duration (Median): 1.4 years		↓ ≈1.0%	↓↓ ≈4.2 kg	↓ Systolic −2.6 (−3.7 to −1.5) mmHg/Diastolic 0.7 (0.0 to 1.3)	Prevent deterioration	Gastrointestinal disorders, ↑ Heart Rate
	Dulaglutide vs. Placebo (REWIND) (22) (29) N = 9901 All diabetic Follow-up duration (Median): 5.4 years	(1) Age ≥ 50 years with ASCVD or unstable angina or cardiac ischemia evident on imaging (2) Age ≥ 55 years with ASCVD (3) Age ≥ 60 years ASCVD + 2 of conditions: tobacco use, dyslipidaemia, hypertension, or abdominal obesity	↓ −0.61%	↓ −1.46 kg (1.25 to 1.67)	↓ −1.70 mmHg (1.33 to 1.07)	Prevent deterioration	Gastrointestinal disorders, ↑ Heart Rate
	Albiglutide vs. Placebo (Harmony Outcomes) (24) (29) N = 9463 All diabetic Follow-up duration (Median): 1.5 years	Age ≥ 40 years with ASCVD and glycated haemoglobin concentration > 7.0% (53 mmol/mole)	↓ −0.52%	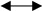 −0.83 kg	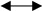 Systolic −0.67 mmHg	Natural effect	Injection site reactions
SGLT2i	Empagliflozin vs. Placebo (EMPA-REG) (47) N = 7020 All diabetic Follow-up duration (Median): 3.1 years	Patients with type 2 diabetes with established ASCVD	↓ −0.54%	↓ −2–3 kg	↓ Systolic −(4–5) mmHg/ Diastolic −(1–2) mmHg	Prevent deterioration	Diabetic ketoacidosis, Genital infection, Urosepsis
	Dapagliflozin vs. Placebo (DECLARE) (50) N = 17,160 All diabetic Follow-up duration (Median): 4.2 years	Age ≥ 40 years with type 2 diabetes, a glycated hemoglobin of 6.5–12.0% and eGFR > 60 mL/min with: (1) ASCVD or (2) multiple risk factors for atherosclerotic cardiovascular disease	↓ 0.42%	↓ 1.8 kg	↓ Systolic −2.7 mmHg (95%/ Diastolic −0.7 mmHg	Prevent deterioration	Diabetic ketoacidosis, Genital infection,
	Canagliflozin vs. Placebo (CANVAS) (49) N = 10,142 All diabetic Follow-up duration (Median): 2.4 years	Age ≥ 30 years with type 2 diabetes, a glycated hemoglobin of ≥7.0% and ≤10.5% with: (1) ASCVD (2) Age > 50 years with two or more of the following: Duration of diabetes of at least 10 years, Systolic blood pressure > 140 mmHg while they were receiving one or more antihypertensive agents, Current smoking, Microalbuminuria or macroalbuminuria High-density lipoprotein cholesterol level of <1 mmol/L (38.7 mg/dL)	↓ −0.58%	↓ −1.60 kg	↓ Systolic −3.93 mmHg/ Diastolic −1.39 mmHg	Prevent deterioration	Diabetic ketoacidosis, Amputation, Fractures, Infection of male genitalia, Mycotic genital infection in women
	Sotagliflozin vs. Placebo (SCORED) (65,66) N = 1222 All diabetic Follow-up duration (Median): 1.3 years	Glycated hemoglobin level of >7%, chronic kidney disease (eGFR, 25 to 60 mL/min/1.73 m^2^), with either: (1) At least one major cardiovascular risk factor (HF, LVEF ≤ 40%, LVH, CAC score > 300 or elevated hsTrop or NT-BNP) (2) Age > 55 years and at least two minor cardiovascular risk factors (BMI > 35, dyslipidemia, smoker, CAC score 100–300, hypertension despite treatment, or positive cardiac family history)	↓ −0.60%	↓ −1.40 K	↓ Systolic −3.54 mmHg/ Diastolic 2.05 mmHg	Natural Effect	Diarrhea, Genital mycotic infections, Diabetic ketoacidosis

Legend: ASCVD definition = Coronary heart disease, cerebrovascular disease, peripheral vascular disease, chronic kidney disease of stage 3 or greater.

**Table 2:** Major clinical outcomes from cardiovascular outcome trials evaluating novel anti-diabetic medications.

	Drug Name	3-Point MACE	Cardiovascular Death	Non-Fatal MI	Non-Fatal Stroke	All-Cause Mortality	Heart Failure Re-Hospitalization
GLP1-RA	Liraglutide LEADER(16)	0.87 (0.78–0.97)	0.78 (0.66–0.93)	0.88 (0.75–1.03)	0.89 (0.72–1.11)	0.85 (0.74–0.97)	0.87 (0.73–1.05)
	Semaglutide SUSTAIN-6 (20)	0.74 (0.58–0.95)	0.98 (0.65–1.48)	0.74 (0.51–1.08)	0.61 (0.38–0.99)	1.05 (0.74–1.50)	1.11 (0.77–1.61)
	Semaglutide (Oral) PIONEER-6 (21)	0.79 (0.57–1.11)	0.49 (0.27–0.92)	1.18 (0.73–1.90)	0.74 (0.35–1.57)	0.51 (0.31–0.84)	0.86 (0.48–1.55)
	Dulaglutide REWIND (22)	0.88 (0.79–0.99)	0.91 (0.78–1.06)	0.96 (0.79–1.16)	0.76 (0.61–0.95)	0.90 (0.80–1.01)	0.93 (0.77–1.12)
	Albiglutide Harmony Outcomes (24)	0.78 (0.68–0.90)	0.93 (0.73–1.19)	Not Reported	Not Reported	0.95 (0.79–1.16)	Not Reported
SGLT2i	Empagliflozin EMPA-REG OUTCOME (47)	0.86 (0.74–0.99)	0.62 (0.49–0.77)	0.87 (0.70–1.09)	1.24 (0.92–1.67)	0.68 (0.57–0.82)	0.65 (0.50–0.85)
	Dapagliflozin DECLARE TIMI-58 (50)	0.93 (0.84–1.03)	0.98 (0.82–1.17)	0.89 (0.77–1.01)	1.01 (0.84–1.21)	0.93 (0.82–1.04)	0.73 (0.61–0.88)
	Canagliflozin CANVAS (49)	0.86 (0.75–0.97)	0.87 (0.72–1.06)	0.85 (0.69–1.05)	0.90 (0.71–1.15)	0.87 (0.74–1.01)	0.67 (0.52–0.87)
	Sotagliflozin SCORED (65)	0.77 (0.65–0.91)	0.90 (0.73–1.12)	Not Reported	Not Reported	0.99 (0.83–1.18)	0.67 (0.55–0.82)
	Significant (*p* < 0.05)			Non-significant			*p* value not reported

See colors in each square: Green—Significant, Orange—Non-significant, Gray—Not reported.

**Table 3:** Major clinical outcomes from heart failure studies evaluating SGLT2i.

	Drug Name	Primary End-Point	Heart Failure Hospitalization	Cardiovascular Death	All Cause Mortality	Worsening Renal Function
HFrEF						
	Empagliflozin EMPEROR-reduced (62)	0.75 (0.65 to 0.86) ^&^	0.69 (0.59 to 0.81)	0.92 (0.75 to 1.12)	0.92 (0.77 to 1.10)	0.50 (0.32 to 0.77)
	Dapagliflozin DAPA-HF (63)	0.74 (0.65 to 0.85) *	0.70 (0.59 to 0.83)	0.82 (0.69 to 0.98)	0.83 (0.71 to 0.97)	0.71 (0.44 to 1.16)
	Sotagliflozin ^$^ SOLOIST-WHF (66)	0.67 (0.52 to 0.85) ^#^	0.64 (0.49 to 0.83)	0.84 (0.58 to 1.22)	0.82 (0.59 to 1.14)	No data
HFpEF						
	Empagliflozin EMPEROR-Preserved (70)	0.79 (0.69 to 0.90) ^&^	0.71 (0.60 to 0.83)	0.91 (0.76 to 1.09)	1.00 (0.87 to 1.15)	1.36 (1.06 to 1.66)
	Dapagliflozin	Ongoing, results expected mid 2022.				
	Significant (*p* < 0.05)					*p* value not re-ported

HFrEF: Heart failure with reduced ejection fraction; HFpEF: Heart failure with preserved ejection fraction. ^&^ The primary outcome was a composite of adjudicated cardiovascular death or hospitalization for heart failure. * The primary end-point was the composite of worsening heart failure (hospitalization or an urgent visit resulting in intravenous therapy for heart failure) or death from cardiovascular causes. ^$^ Trial included both HFrEF and HFpEF patients. ^#^ Primary end-point included death from cardiovascular causes and hospitalizations and urgent visits for heart failure—total number of events.

## Abbreviations

ASCVD	Atherosclerotic cardiovascular disease
CVD	Cardiovascular disease
CVOTs	Cardiovascular outcome trials
DM	Diabetes mellitus
DPP-4	Dipeptidyl peptidase-4
GLP-1	Glucagon-like peptide-1
GLP-1RA	Glucagon-like peptide-1 receptor agonists
HFrEF	Heart Failure with reduced ejection fraction
HFpEF	Heart Failure with Preserved ejection fraction
MACE	Major adverse cardiovascular events
MI	Myocardial infarction
SGLT2i	Sodium-glucose cotransporter 2 inhibitor

## References

[1] Zimmet P.Z., Magliano D.J., Herman W.H., Shaw J.E. (2014). Diabetes: A 21st Century Challenge. Lanect Diabetes Endocrinol..

[2] Cho N.H., Shaw J.E., Karuranga S., Huang Y., da Rocha Fernandes J.D., Ohlrogge A.W., Malanda B. (2018). IDF Diabetes Atlas: Global Estimates of Diabetes Prevalence for 2017 and Projections for 2045. Diabetes Res. Clin. Pract..

[3] Lawrence J.M., Divers J., Isom S., Saydah S., Imperatore G., Pihoker C., Marcovina S.M., Mayer-Davis E.J., Hamman R.F., Dolan L. (2021). Trends in Prevalence of Type 1 and Type 2 Diabetes in Children and Adolescents in the US, 2001–2017. JAMA.

[4] Timmis A., Townsend N., Gale C.P., Torbica A., Lettino M., Petersen S.E., Mossialos E.A., Maggioni A.P., Kazakiewicz D., May H.T. (2019). European Society of Cardiology: Cardiovascular Disease Statistics 2019. Eur. Heart J..

[5] Holman R.R., Paul S.K., Bethel M.A., Matthews D.R., Neil H.A.W. (2008). 10-Year Follow-Up of Intensive Glucose Control in Type 2 Diabetes. N. Engl. J. Med..

[6] Diabetes Control and Complications Trial Research Group (1993). The Effect of Intensive Treatment of Diabetes on the Development and Progression of Long-Term Complications in Insulin-Dependent Diabetes Mellitus. N. Engl. J. Med..

[7] Gregg E.W., Cheng Y.J., Srinivasan M., Lin J., Geiss L.S., Albright A.L., Imperatore G. (2018). Trends in Cause-Specific Mortality among Adults with and without Diagnosed Diabetes in the USA: An Epidemiological Analysis of Linked National Survey and Vital Statistics Data. Lancet.

[8] Collaboration E.R.F., Sarwar N., Gao P., Seshasai S.R.K., Gobin R., Kaptoge S., Angelantonio E.D., Ingelsson E., Lawlor D.A., Selvin E. (2010). Diabetes Mellitus, Fasting Blood Glucose Concentration, and Risk of Vascular Disease: A Collaborative Meta-Analysis of 102 Prospective Studies. Lancet.

[9] Rawshani A., Rawshani A., Franzén S., Eliasson B., Svensson A.-M., Miftaraj M., McGuire D.K., Sattar N., Rosengren A., Gudbjörnsdottir S. (2017). Mortality and Cardiovascular Disease in Type 1 and Type 2 Diabetes. N. Engl. J. Med..

[10] Arnett D.K., Blumenthal R.S., Albert M.A., Buroker A.B., Goldberger Z.D., Hahn E.J., Himmelfarb C.D., Khera A., Lloyd-Jones D., McEvoy J.W. (2019). 2019 ACC/AHA Guideline on the Primary Prevention of Cardiovascular Disease. Circulation.

[11] Visseren F.L.J., Mach F., Smulders Y.M., Carballo D., Koskinas K.C., Bäck M., Benetos A., Biffi A., Boavida J.-M., Capodanno D. (2021). 2021 ESC Guidelines on Cardiovascular Disease Prevention in Clinical PracticeDeveloped by the Task Force for Cardiovascular Disease Prevention in Clinical Practice with Representatives of the European Society of Cardiology and 12 Medical Societies with the Special Contribution of the European Association of Preventive Cardiology (EAPC). Eur. Heart J..

[12] Newman J.D., Vani A.K., Aleman J.O., Weintraub H.S., Berger J.S., Schwartzbard A.Z. (2018). The Changing Landscape of Diabetes Therapy for Cardiovascular Risk Reduction JACC State-of-the-Art Review. J. Am. Coll. Cardiol..

[13] Pálsson R., Patel U.D. (2014). Cardiovascular Complications of Diabetic Kidney Disease. Adv. Chronic. Kidney Dis..

[14] Nauck M.A., Vardarli I., Deacon C.F., Holst J.J., Meier J.J. (2011). Secretion of Glucagon-like Peptide-1 (GLP-1) in Type 2 Diabetes: What Is up, What Is Down?. Diabetologia.

[15] Nauck M.A. (2020). The Rollercoaster History of Using Physiological and Pharmacological Properties of Incretin Hormones to Develop Diabetes Medications with a Convincing Benefit-Risk Relationship. Metab. Clin. Exp..

[16] Marso S.P., Daniels G.H., Brown-Frandsen K., Kristensen P., Mann J.F.E., Nauck M.A., Nissen S.E., Pocock S., Poulter N.R., Ravn L.S. (2016). Liraglutide and Cardiovascular Outcomes in Type 2 Diabetes. N. Engl. J. Med..

[17] Crowley M.J., McGuire D.K., Alexopoulos A.-S., Jensen T.J., Rasmussen S., Saevereid H.A., Verma S., Buse J.B. (2020). Effects of Liraglutide on Cardiovascular Outcomes in Type 2 Diabetes Patients with and without Baseline Metformin Use: Post Hoc Analyses of the LEADER Trial. Diabetes Care.

[18] Verma S., Bain S.C., Buse J.B., Idorn T., Rasmussen S., Ørsted D.D., Nauck M.A. (2019). Occurence of First and Recurrent Major Adverse Cardiovascular Events with Liraglutide Treatment Among Patients with Type 2 Diabetes and High Risk of Cardiovascular Events. JAMA Cardiol..

[19] Svanström H., Ueda P., Melbye M., Eliasson B., Svensson A.-M., Franzén S., Gudbjörnsdottir S., Hveem K., Jonasson C., Pasternak B. (2019). Use of Liraglutide and Risk of Major Cardiovascular Events: A Register-Based Cohort Study in Denmark and Sweden. Lancet Diabetes Endocrinol..

[20] Marso S.P., Bain S.C., Consoli A., Eliaschewitz F.G., Jódar E., Leiter L.A., Lingvay I., Rosenstock J., Seufert J., Warren M.L. (2016). Semaglutide and Cardiovascular Outcomes in Patients with Type 2 Diabetes. N. Engl. J. Med..

[21] Husain M., Birkenfeld A.L., Donsmark M., Dungan K., Eliaschewitz F.G., Franco D.R., Jeppesen O.K., Lingvay I., Mosenzon O., Pedersen S.D. (2019). Oral Semaglutide and Cardiovascular Outcomes in Patients with Type 2 Diabetes. N. Engl. J. Med..

[22] Gerstein H.C., Colhoun H.M., Dagenais G.R., Diaz R., Lakshmanan M., Pais P., Probstfield J., Riesmeyer J.S., Riddle M.C., Rydén L. (2019). Dulaglutide and Cardiovascular Outcomes in Type 2 Diabetes (REWIND): A Double-Blind, Randomised Placebo-Controlled Trial. Lancet.

[23] Tahrani A.A., Barnett A.H., Bailey C.J. (2016). Pharmacology and Therapeutic Implications of Current Drugs for Type 2 Diabetes Mellitus. Nat. Rev. Endocrinol..

[24] Hernandez A.F., Green J.B., Janmohamed S., D’Agostino R.B., Granger C.B., Jones N.P., Leiter L.A., Rosenberg A.E., Sigmon K.N., Somerville M.C. (2018). Albiglutide and Cardiovascular Outcomes in Patients with Type 2 Diabetes and Cardiovascular Disease (Harmony Outcomes): A Double-Blind, Randomised Placebo-Controlled Trial. Lancet.

[25] Zhang Z., Chen X., Lu P., Zhang J., Xu Y., He W., Li M., Zhang S., Jia J., Shao S. (2017). Incretin-Based Agents in Type 2 Diabetic Patients at Cardiovascular Risk: Compare the Effect of GLP-1 Agonists and DPP-4 Inhibitors on Cardiovascular and Pancreatic Outcomes. Cardiovasc. Diabetol..

[26] Lin D.S.-H., Lee J.-K., Chen W.-J. (2021). Major Adverse Cardiovascular and Limb Events in Patients with Diabetes Treated with GLP-1 Receptor Agonists vs DPP-4 Inhibitors. Diabetologia.

[27] Longato E., Camillo B.D., Sparacino G., Tramontan L., Avogaro A., Fadini G.P. (2020). Better Cardiovascular Outcomes of Type 2 Diabetic Patients Treated with GLP-1 Receptor Agonists versus DPP-4 Inhibitors in Clinical Practice. Cardiovasc. Diabetol..

[28] Anyanwagu U., Mamza J., Donnelly R., Idris I. (2018). Effect of Adding GLP-1RA on Mortality, Cardiovascular Events, and Metabolic Outcomes among Insulin-Treated Patients with Type 2 Diabetes: A Large Retrospective UK Cohort Study. Am. Heart J..

[29] Kristensen S.L., Rørth R., Jhund P.S., Docherty K.F., Sattar N., Preiss D., Køber L., Petrie M.C., McMurray J.J.V. (2019). Cardiovascular, Mortality, and Kidney Outcomes with GLP-1 Receptor Agonists in Patients with Type 2 Diabetes: A Systematic Review and Meta-Analysis of Cardiovascular Outcome Trials. Lancet Diabetes Endocrinol..

[30] Baigent C., Blackwell L., Emberson J., Holland L.E., Reith C., Bhala N., Peto R., Barnes E.H., Keech A., Cholesterol Treatment Trialists’ (CTT) Collaboration (2010). Efficacy and Safety of More Intensive Lowering of LDL Cholesterol: A Meta-Analysis of Data from 170,000 Participants in 26 Randomised Trials. Lancet.

[31] Rakipovski G., Rolin B., Nøhr J., Klewe I., Frederiksen K.S., Augustin R., Hecksher-Sørensen J., Ingvorsen C., Polex-Wolf J., Knudsen L.B. (2018). The GLP-1 Analogs Liraglutide and Semaglutide Reduce Atherosclerosis in ApoE−/− and LDLr−/− Mice by a Mechanism That Includes Inflammatory Pathways. JACC Basic. Trans. Sci..

[32] Anholm C., Kumarathurai P., Pedersen L.R., Samkani A., Walzem R.L., Nielsen O.W., Kristiansen O.P., Fenger M., Madsbad S., Sajadieh A. (2019). Liraglutide in Combination with Metformin May Improve the Atherogenic Lipid Profile and Decrease C-Reactive Protein Level in Statin Treated Obese Patients with Coronary Artery Disease and Newly Diagnosed Type 2 Diabetes: A Randomized Trial. Atherosclerosis.

[33] Nikolic D., Giglio R.V., Rizvi A.A., Patti A.M., Montalto G., Maranta F., Cianflone D., Stoian A.P., Rizzo M. (2021). Liraglutide Reduces Carotid Intima-Media Thickness by Reducing Small Dense Low-Density Lipoproteins in a Real-World Setting of Patients with Type 2 Diabetes: A Novel Anti-Atherogenic Effect. Diabetes Ther..

[34] Ma X., Liu Z., Ilyas I., Little P.J., Kamato D., Sahebka A., Chen Z., Luo S., Zheng X., Weng J. (2021). GLP-1 Receptor Agonists (GLP-1RAs): Cardiovascular Actions and Therapeutic Potential. Int. J. Biol. Sci..

[35] Shaman A.M., Bain S.C., Bakris G.L., Buse J.B., Idorn T., Mahaffey K.W., Mann J.F.E., Nauck M.A., Rasmussen S., Rossing P. (2021). Effect of the Glucagon-like Peptide-1 Receptor Agonists Semaglutide and Liraglutide on Kidney Outcomes in Patients with Type 2 Diabetes: A Pooled Analysis of SUSTAIN 6 and LEADER Trials. Circulation.

[36] Aziz M.A.E., Cahyadi O., Meier J.J., Schmidt W.E., Nauck M.A. (2020). Incretin-Based Glucose-Lowering Medications and the Risk of Acute Pancreatitis and Malignancies: A Meta-Analysis Based on Cardiovascular Outcomes Trials. Diabetes Obes. Metab..

[37] Cao C., Yang S., Zhou Z. (2020). GLP-1 Receptor Agonists and Pancreatic Safety Concerns in Type 2 Diabetic Patients: Data from Cardiovascular Outcome Trials. Endocrine.

[38] Knudsen L.B., Madsen L.W., Andersen S., Almholt K., Boer A.S., Drucker D.J., Gotfredsen C., Egerod F.L., Hegelund A.C., Jacobsen H. (2010). Glucagon-like Peptide-1 Receptor Agonists Activate Rodent Thyroid C-Cells Causing Calcitonin Release and C-Cell Proliferation. Endocrinology.

[39] Aroda V.R., Bain S.C., Cariou B., Piletič M., Rose L., Axelsen M., Rowe E., DeVries J.H. (2017). Efficacy and Safety of Once-Weekly Semaglutide versus Once-Daily Insulin Glargine as Add-on to Metformin (with or without Sulfonylureas) in Insulin-Naive Patients with Type 2 Diabetes (SUSTAIN 4): A Randomised, Open-Label, Parallel-Group, Multicentre, Multinational, Phase 3a Trial. Lancet Diabetes Endocrinol..

[40] Rieg T., Vallon V. (2018). Development of SGLT1 and SGLT2 Inhibitors. Diabetologia.

[41] Pasternak B., Ueda P., Eliasson B., Svensson A.M., Franzén S., Gudbjörnsdottir S., Hveem K., Jonasson C., Wintzell V., Melbye M. (2019). Use of Sodium Glucose Cotransporter 2 Inhibitors and Risk of Major Cardiovascular Events and Heart Failure: Scandinavian Register Based Cohort Study. BMJ.

[42] Xie Y., Bowe B., Gibson A.K., McGill J.B., Maddukuri G., Al-Aly Z. (2021). Comparative Effectiveness of Sodium-Glucose Cotransporter 2 Inhibitors vs Sulfonylureas in Patients with Type 2 Diabetes. JAMA Int. Med..

[43] Kosiborod M., Lam C.S.P., Kohsaka S., Kim D.J., Karasik A., Shaw J., Tangri N., Goh S.-Y., Thuresson M., Chen H. (2018). Cardiovascular Events Associated with SGLT-2 Inhibitors versus Other Glucose-Lowering Drugs The CVD-REAL 2 Study. J. Am. Coll. Cardiol..

[44] Real J., Vlacho B., Ortega E., Vallés J.A., Mata-Cases M., Castelblanco E., Wittbrodt E.T., Fenici P., Kosiborod M., Mauricio D. (2021). Cardiovascular and Mortality Benefits of Sodium–Glucose Co-Transporter-2 Inhibitors in Patients with Type 2 Diabetes Mellitus: CVD-Real Catalonia. Cardiovasc. Diabetol..

[45] Jeon J.Y., Ha K.H., Kim D.J. (2020). Cardiovascular Safety of Sodium Glucose Cotransporter 2 Inhibitors as Add-on to Metformin Monotherapy in Patients with Type 2 Diabetes Mellitus. Korean Diabetes J..

[46] Kosiborod M., Cavender M.A., Fu A.Z., Wilding J.P., Khunti K., Holl R.W., Norhammar A., Birkeland K.I., Jørgensen M.E., Thuresson M. (2017). Lower Risk of Heart Failure and Death in Patients Initiated on Sodium-Glucose Cotransporter-2 Inhibitors versus Other Glucose-Lowering Drugs. Circulation.

[47] Zinman B., Wanner C., Lachin J.M., Fitchett D., Bluhmki E., Hantel S., Mattheus M., Devins T., Johansen O.E., Woerle H.J. (2015). Empagliflozin, Cardiovascular Outcomes, and Mortality in Type 2 Diabetes. N. Engl. J. Med..

[48] McGuire D.K., Zinman B., Inzucchi S.E., Wanner C., Fitchett D., Anker S.D., Pocock S., Kaspers S., George J.T., von Eynatten M. (2020). Effects of Empagliflozin on First and Recurrent Clinical Events in Patients with Type 2 Diabetes and Atherosclerotic Cardiovascular Disease: A Secondary Analysis of the EMPA-REG OUTCOME Trial. Lancet Diabetes Endocrinol..

[49] Neal B., Perkovic V., Mahaffey K.W., De Zeeuw D., Fulcher G., Erondu N., Shaw W., Law G., Desai M., Matthews D.R. (2017). Canagliflozin and Cardiovascular and Renal Events in Type 2 Diabetes. N. Engl. J. Med..

[50] Wiviott S.D., Raz I., Bonaca M.P., Mosenzon O., Kato E.T., Cahn A., Silverman M.G., Zelniker T.A., Kuder J.F., Murphy S.A. (2019). Dapagliflozin and Cardiovascular Outcomes in Type 2 Diabetes. N. Engl. J. Med..

[51] Cosentino F., Grant P.J., Aboyans V., Bailey C.J., Ceriello A., Delgado V., Federici M., Filippatos G., Grobbee D.E., Hansen T.B. (2019). 2019 ESC Guidelines on Diabetes, Pre-Diabetes, and Cardiovascular Diseases Developed in Collaboration with the EASD. Eur. Heart J..

[52] McMurray J.J.V., Solomon S.D., Inzucchi S.E., Køber L., Kosiborod M.N., Martinez F.A., Ponikowski P., Sabatine M.S., Anand I.S., Bělohlávek J. (2019). Dapagliflozin in Patients with Heart Failure and Reduced Ejection Fraction. N. Engl. J. Med..

[53] Packer M., Anker S.D., Butler J., Filippatos G., Pocock S.J., Carson P., Januzzi J., Verma S., Tsutsui H., Brueckmann M. (2020). Cardiovascular and Renal Outcomes with Empagliflozin in Heart Failure. N. Engl. J. Med..

[54] Baker W.L., Smyth L.R., Riche D.M., Bourret E.M., Chamberlin K.W., White W.B. (2014). Effects of Sodium-Glucose Co-Transporter 2 Inhibitors on Blood Pressure: A Systematic Review and Meta-Analysis. J. Am. Soc. Hypertens..

[55] Heerspink H.J.L., Perkins B.A., Fitchett D.H., Husain M., Cherney D.Z.I. (2016). Sodium Glucose Cotransporter 2 Inhibitors in the Treatment of Diabetes Mellitus. Circulation.

[56] Verma S., Rawat S., Ho K.L., Wagg C.S., Zhang L., Teoh H., Dyck J.E., Uddin G.M., Oudit G.Y., Mayoux E. (2018). Empagliflozin Increases Cardiac Energy Production in Diabetes: Novel Translational Insights Into the Heart Failure Benefits of SGLT2 Inhibitors. JACC Basic Trans. Sci..

[57] Goonasekera S.A., Hammer K., Auger-Messier M., Bodi I., Chen X., Zhang H., Reiken S., Elrod J.W., Correll R.N., York A.J. (2012). Decreased Cardiac L-Type Ca2+ Channel Activity Induces Hypertrophy and Heart Failure in Mice. J. Clin. Investig..

[58] Hawley S.A., Ford R.J., Smith B.K., Gowans G.J., Mancini S.J., Pitt R.D., Day E.A., Salt I.P., Steinberg G.R., Hardie D.G. (2016). The Na+/Glucose Cotransporter Inhibitor Canagliflozin Activates AMPK by Inhibiting Mitochondrial Function and Increasing Cellular AMP Levels. Diabetes.

[59] Zhou H., Wang S., Zhu P., Hu S., Chen Y., Ren J. (2018). Empagliflozin Rescues Diabetic Myocardial Microvascular Injury via AMPK-Mediated Inhibition of Mitochondrial Fission. Redox Biol..

[60] Packer M. (2017). Activation and Inhibition of Sodium-Hydrogen Exchanger Is a Mechanism That Links the Pathophysiology and Treatment of Diabetes Mellitus with That of Heart Failure. Circulation.

[61] Zannad F., Ferreira J.P., Pocock S.J., Anker S.D., Butler J., Filippatos G., Brueckmann M., Ofstad A.P., Pfarr E., Jamal W. (2020). SGLT2 Inhibitors in Patients with Heart Failure with Reduced Ejection Fraction: A Meta-Analysis of the EMPEROR-Reduced and DAPA-HF Trials. Lancet.

[62] Packer M., Anker S.D., Butler J., Filippatos G., Ferreira J.P., Pocock S.J., Carson P., Anand I., Doehner W., Haass M. (2021). Effect of Empagliflozin on the Clinical Stability of Patients with Heart Failure and a Reduced Ejection Fraction: The EMPEROR-Reduced Trial. Circulation.

[63] Berg D.D., Jhund P.S., Docherty K.F., Murphy S.A., Verma S., Inzucchi S.E., Køber L., Kosiborod M.N., Langkilde A.M., Martinez F.A. (2021). Time to Clinical Benefit of Dapagliflozin and Significance of Prior Heart Failure Hospitalization in Patients with Heart Failure with Reduced Ejection Fraction. JAMA Cardiol..

[64] Rao V.N., Murray E., Butler J., Cooper L.B., Cox Z.L., Fiuzat M., Green J.B., Lindenfeld J., McGuire D.K., Nassif M.E. (2021). In-Hospital Initiation of Sodium-Glucose Cotransporter-2 Inhibitors for Heart Failure with Reduced Ejection Fraction. J. Am. Coll. Cardiol..

[65] Powell D.R., Zambrowicz B., Morrow L., Beysen C., Hompesch M., Turner S., Hellerstein M., Banks P., Strumph P., Lapuerta P. (2019). Sotagliflozin Decreases Postprandial Glucose and Insulin Concentrations by Delaying Intestinal Glucose Absorption. J. Clin. Endocrinol. Metab..

[66] Bhatt D.L., Szarek M., Pitt B., Cannon C.P., Leiter L.A., McGuire D.K., Lewis J.B., Riddle M.C., Inzucchi S.E., Kosiborod M.N. (2020). Sotagliflozin in Patients with Diabetes and Chronic Kidney Disease. N. Engl. J. Med..

[67] Wright E.M., Loo D.D.F., Hirayama B.A. (2011). Biology of Human Sodium Glucose Transporters. Physiol. Rev..

[68] Bhatt D.L., Szarek M., Steg P.G., Cannon C.P., Leiter L.A., McGuire D.K., Lewis J.B., Riddle M.C., Voors A.A., Metra M. (2020). Sotagliflozin in Patients with Diabetes and Recent Worsening Heart Failure. N. Engl. J. Med..

[69] Kato E.T., Silverman M.G., Mosenzon O., Zelniker T.A., Cahn A., Furtado R.H.M., Kuder J., Murphy S.A., Bhatt D.L., Leiter L.A. (2019). Effect of Dapagliflozin on Heart Failure and Mortality in Type 2 Diabetes Mellitus. Circulation.

[70] Anker S.D., Butler J., Filippatos G., Ferreira J.P., Bocchi E., Böhm M., Rocca H.-P.B.-L., Choi D.-J., Chopra V., Chuquiure-Valenzuela E. (2021). Empagliflozin in Heart Failure with a Preserved Ejection Fraction. N. Engl. J. Med..

[71] Nassif M.E., Windsor S.L., Borlaug B.A., Kitzman D.W., Shah S.J., Tang F., Khariton Y., Malik A.O., Khumri T., Umpierrez G. (2021). The SGLT2 Inhibitor Dapagliflozin in Heart Failure with Preserved Ejection Fraction: A Multicenter Randomized Trial. Nat. Med..

[72] Butler J., Filippatos G., Siddiqi T.J., Brueckmann M., Böhm M., Chopra V., Ferreira J.P., Januzzi J.L., Kaul S., Piña I.L. (2021). Empagliflozin, Health Status, and Quality of Life in Patients with Heart Failure and Preserved Ejection Fraction: The EMPEROR-Preserved Trial. Circulation.

[73] Solomon S.D., Boer R.A., DeMets D., Hernandez A.F., Inzucchi S.E., Kosiborod M.N., Lam C.S.P., Martinez F., Shah S.J., Lindholm D. (2021). Dapagliflozin in Heart Failure with Preserved and Mildly Reduced Ejection Fraction: Rationale and Design of the DELIVER Trial. Eur. J. Heart Fail..

[74] Damman K., Beusekamp J.C., Boorsma E.M., Swart H.P., Smilde T.D.J., Elvan A., Eck J.W.M., Heerspink H.J.L., Voors A.A. (2020). Randomized, Double-blind, Placebo-controlled, Multicentre Pilot Study on the Effects of Empagliflozin on Clinical Outcomes in Patients with Acute Decompensated Heart Failure (EMPA-RESPONSE-AHF). Eur. J. Heart Fail..

[75] Voors A.A., Angermann C.E., Teerlink J.R., Collins S.P., Kosiborod M., Biegus J., Ferreira J.P., Nassif M.E., Psotka M.A., Tromp J. (2022). The SGLT2 Inhibitor Empagliflozin in Patients Hospitalized for Acute Heart Failure: A Multinational Randomized Trial. Nat. Med..

[76] Wanner C., Inzucchi S.E., Lachin J.M., Fitchett D., Eynatten M., von Mattheus M., Johansen O.E., Woerle H.J., Broedl U.C., Zinman B. (2016). Empagliflozin and Progression of Kidney Disease in Type 2 Diabetes. N. Engl. J. Med..

[77] Heerspink H.J.L., Stefánsson B.V., Correa-Rotter R., Chertow G.M., Greene T., Hou F.-F., Mann J.F.E., McMurray J.J.V., Lindberg M., Rossing P. (2020). Dapagliflozin in Patients with Chronic Kidney Disease. N. Engl. J. Med..

[78] McGill J.B., Subramanian S. (2019). Safety of Sodium-Glucose Co-Transporter 2 Inhibitors. Am. J. Cardiol..

[79] Fadini G.P., Bonora B.M., Avogaro A. (2017). SGLT2 Inhibitors and Diabetic Ketoacidosis: Data from the FDA Adverse Event Reporting System. Diabetologia.

[80] Association D. (2020). Addendum. 10. Cardiovascular Disease and Risk Management: Standards of Medical Care in Diabetes—2021. Diabetes Care.

[81] Committee W., Das S.R., Everett B.M., Birtcher K.K., Brown J.M., Januzzi J.L., Kalyani R.R., Kosiborod M., Magwire M., Morris P.B. (2020). 2020 Expert Consensus Decision Pathway on Novel Therapies for Cardiovascular Risk Reduction in Patients with Type 2 Diabetes A Report of the American College of Cardiology Solution Set Oversight Committee. J. Am. Coll. Cardiol..

[82] McDonagh T.A., Metra M., Adamo M., Gardner R.S., Baumbach A., Böhm M., Burri H., Butler J., Čelutkienė J., Chioncel O. (2021). 2021 ESC Guidelines for the Diagnosis and Treatment of Acute and Chronic Heart FailureDeveloped by the Task Force for the Diagnosis and Treatment of Acute and Chronic Heart Failure of the European Society of Cardiology (ESC) with the Special Contribution of the Heart Failure Association (HFA) of the ESC. Eur. Heart J..

[83] Committee W., Maddox T.M., Januzzi J.L., Allen L.A., Breathett K., Butler J., Davis L.L., Fonarow G.C., Ibrahim N.E., Lindenfeld J. (2021). 2021 Update to the 2017 ACC Expert Consensus Decision Pathway for Optimization of Heart Failure Treatment: Answers to 10 Pivotal Issues About Heart Failure with Reduced Ejection Fraction: A Report of the American College of Cardiology Solution Set Oversight Committee. J. Am. Coll. Cardiol..

[84] American Diabetes Association (2015). 3. Foundations of Care and Comprehensive Medical Evaluation. Diabetes Care.

[85] Association A.D. (2015). 6. Obesity Management for the Treatment of Type 2 Diabetes. Diabetes Care.

[86] Jarvie J.L., Whooley M.A., Regan M.C., Sin N.L., Cohen B.E. (2014). Effect of Physical Activity Level on Biomarkers of Inflammation and Insulin Resistance Over 5 Years in Outpatients with Coronary Heart Disease (from the Heart and Soul Study). Am. J. Cardiol..

[87] Ross R., Janssen I., Dawson J., Kungl A., Kuk J.L., Wong S.L., Nguyen-Duy T., Lee S., Kilpatrick K., Hudson R. (2004). Exercise-Induced Reduction in Obesity and Insulin Resistance in Women: A Randomized Controlled Trial. Obes. Res..

[88] Ståhle A., Mattsson E., Rydén L., Unden A.-L., Nordlander R. (1999). Improved Physical Fitness and Quality of Life Following Training of Elderly Patients after Acute Coronary Events. A 1 Year Follow-up Randomized Controlled Study. Eur. Heart J..

[89] Sattelmair J., Pertman J., Ding E.L., Kohl H.W., Haskell W., Lee I.-M. (2011). Dose Response Between Physical Activity and Risk of Coronary Heart Disease. Circulation.

[90] Wei M., Kampert J.B., Barlow C.E., Nichaman M.Z., Gibbons L.W., Paffenbarger J.R.S., Blair S.N. (1999). Relationship Between Low Cardiorespiratory Fitness and Mortality in Normal-Weight, Overweight, and Obese Men. JAMA.

[91] Shah R.V., Murthy V.L., Colangelo L.A., Reis J., Venkatesh B.A., Sharma R., Abbasi S.A., Goff D.C., Carr J.J., Rana J.S. (2015). Association of Fitness in Young Adulthood with Survival and Cardiovascular Risk: The Coronary Artery Risk Development in Young Adults (CARDIA) Study. JAMA Int. Med..

[92] Pandey A., Garg S., Khunger M., Darden D., Ayers C., Kumbhani D.J., Mayo H.G., de Lemos J.A., Berry J.D. (2015). Dose–Response Relationship Between Physical Activity and Risk of Heart Failure. Circulation.

[93] Ryan D.H., Lingvay I., Colhoun H.M., Deanfield J., Emerson S.S., Kahn S.E., Kushner R.F., Marso S., Plutzky J., Brown-Frandsen K. (2020). Semaglutide Effects on Cardiovascular Outcomes in People with Overweight or Obesity (SELECT) Rationale and Design. Am. Heart J..

[94] Braunwald E. (2021). SGLT2 Inhibitors: The Statins of the 21st Century. Eur. Heart J..

